# Profile of risk factors for people who inject drugs among a sample at high risk of HIV/AIDS in Kigali, Rwanda

**DOI:** 10.3389/adar.2026.16156

**Published:** 2026-10-01

**Authors:** Samuel Habimana, Zephon Lister, Emmanuel Biracyaza, Aflodis Kagaba, Albert Ndagijimana, Stefan Jansen, Eugene Rutembesa, Susanne Montgomery

**Affiliations:** 1 School of Behavioral Health, Loma Linda University, Loma Linda, CA, United States; 2 Rwanda Resilience and Grounding Organization (RRGO), Kigali, Rwanda; 3 School of Rehabilitation, Faculty of Medicine, Université de Montréal, Montréal, QC, Canada; 4 Centre for Interdisciplinary Research in Rehabilitation of Greater Montreal (CRIR), Montreal, QC, Canada; 5 Health Development Initiatives (HDI), Kigali, Rwanda; 6 School of Public Health, University of Rwanda, Kigali, Rwanda; 7 Research and Innovation Center, University of Rwanda, Kigali, Rwanda; 8 Department of Clinical Psychology, University of Rwanda, Kigali, Rwanda

**Keywords:** female sex workers, factors, health development initiatives, HIV/AIDS, Kigali, men who have sex with men, people who inject drugs

## Abstract

**Background:**

The increasing prevalence of people who inject drugs (PWID) among populations at high risk for human immunodeficiency virus (HIV) has become a critical public health concern, particularly in Africa. This issue is associated with substantial health-related effects among populations at high risk for HIV/AIDS. Hence, our study aimed to analyze the associations between sociodemographic characteristics, alcohol and substance dependence, and depressive symptoms among populations at high risk for HIV.

**Methods:**

We conducted a cross-sectional study with 480 respondents living in Kigali, Rwanda, who self-identified as being at high risk for HIV/AIDS and were recruited via snowball sampling. Chi-square bivariate analyses were used to explore associations with PWID status. Variables that were significant in the bivariate analyses were included in a multivariate logistic regression model, in which adjusted odds ratios were used to determine factors associated with PWID status. A 95% confidence interval and a 5% significance level were used to evaluate statistical associations.

**Results:**

Of the 480 respondents, 86.25% reported symptoms of depression, and 31.5% were identified as PWID. Notably, 78% were male and were between 18 years and 45 years of age. Multivariate regression analysis revealed that participants with substance dependence [aOR = 3.547; 95% CI (1.159–10.857)] and those who used marijuana [aOR = 3.261; 95% CI (1.380–7.708)] were more likely to be PWID than their counterparts. Furthermore, individuals with depressive symptoms [aOR = 4.50; 95% CI (2.55–7.96), p = 0.018] were also more likely to be PWID than their counterparts.

**Conclusion:**

The results of this study indicated that substance use and alcohol-related behaviors, as well as mental health concerns such as depressive symptoms, were significantly associated with injecting drug use. The findings support the promotion of harm-reduction and prevention strategies that incorporate mental health programs for populations at high risk for HIV.

## Introduction

Worldwide, drug use and drug dependence are increasing significantly, with 296 million people using drugs globally [1]. This represents a 23% increase over the past decade. Most people who use drugs (PWUD), approximately 78%, reside in low- and middle-income countries and are concentrated predominantly in urban settings [2, 3]. Moreover, among people who use drugs, the number of individuals with a drug use disorder increased by more than 12 million in 2022 [4]. Substance use and drug dependence therefore pose significant global challenges, particularly because they are often associated with crime and place additional burdens on already strained public health services. Globally, approximately 11 million people have engaged in injecting drug use, and the prevalence of PWID has shown a consistent upward trend since 2017 across various regions. In Latin America, estimates range from 1,392,000 to 2,380,000; in Central Asia, from 400,000 to 510,000 [5]; and in South Asia, from 783,500 to 1,263,000. These growing numbers underscore the expanding impact of addictive behaviors across countries, with low- and middle-income countries being especially affected [5, 6]. Moreover, approximately 1 in 8 PWID (1.4 million) are living with HIV (UNODC World Drug Report, 2020), while 39.4% have viremic HCV infection [7]. Populations at high risk for HIV/AIDS include PWID, men who have sex with men (MSM), and female sex workers (FSW). These groups continue to contribute to the burden of HIV/AIDS, emphasizing the need for immediate, targeted interventions that address the distinct needs of each population [5].

Geographically, the prevalence of PWID is relatively higher in towns and urban areas than in rural areas [2]. Although African countries have historically reported low rates of injecting drug use, this pattern has shifted over the past 25 years, with heroin injection increasing in large towns in Kenya, Uganda, and Tanzania since 2009 [8]. The prevalence of injecting drug use has notably increased in East African countries, particularly Tanzania, Kenya, and Rwanda. This increase has had detrimental effects on quality of life and has increased the risk of blood-borne diseases and sexually transmitted infections, including HIV [9–12]. Notably, vulnerability to HIV is pronounced among groups such as MSM, FSW, and PWID, who currently exhibit high HIV prevalence [11, 13, 14].

The relationship between PWID and HIV/AIDS is well established, as surveys in India and China have demonstrated links between HIV/AIDS and drug use, including alcohol and other substances [6, 15]. Currently, the association between HIV/AIDS and injecting drug use remains an important public health concern, particularly given the high HIV prevalence among these populations [9, 11, 13, 14].

Many factors have been identified as increasing HIV/AIDS risk among this population. For example, sharing injecting equipment among PWID serves as a significant route for the transmission of HIV and other blood-borne diseases. A comprehensive systematic review that screened 55,671 papers and reports and extracted data from 1,147 eligible records documented substantial evidence of injecting drug use [2, 5]. This phenomenon was documented in 179 of 206 countries or territories, covering 99% of the population aged 15 years–64 years. According to the Centers for Disease Control and Prevention (CDC, 2021), approximately 1 in 10 new HIV infections can be attributed to injecting drug use [16]. These findings underscore the global impact of injecting drug use on HIV transmission and emphasize the urgent need for targeted interventions and public health initiatives [16].

According to the preceding literature, PWID can be influenced by a multitude of interconnected factors [2]. Factors associated with injection drug use encompass a range of experiences, from engagement in sex work and a history of sexual abuse to exposure to trauma and violence, social disadvantage, family history of drug use, and incarceration [17–19]. Social environments play a significant role, as peer pressure and the prevalence of drug use within communities can sway individuals toward injecting drugs. Mental health also plays a pivotal role, with conditions such as depression, previous substance use, trauma, adverse childhood experiences, anxiety, or trauma leading some to seek solace in substances [20]. Genetics may contribute, as certain individuals might be more genetically predisposed to addiction (NIDA, 2023). Access to drugs, whether through social circles or easy availability in illegal markets, can increase the likelihood of drug use. Socioeconomic factors, including poverty and a lack of opportunities, might drive individuals toward substances as a means of escape [17, 18]. Additionally, cultural influences and media representations can normalize or glamorize drug use, potentially influencing individuals’ ability to experiment with or resort to injecting drugs [19].

Depression has emerged as a widespread public health concern globally [21]. However, rates of depression vary across communities, with an increasing trend observed among PWID in several East African countries, including Kenya [22]. In Rwanda, depression affects 35% of genocide survivors and 20% of the general population [23]. Additionally, individuals living with HIV/AIDS in Rwanda experience depression, which is often associated with stigma and medication nonadherence [24]. Some individuals experiencing depression or other mental health concerns may turn to substance use, including injecting drugs, as a means of coping with emotional distress or self-medicating [25].

Harm-reduction or risk-minimization strategies targeting the behaviors of PWID in Rwanda have received limited attention and promotion. Recent research by Twahirwa Rwema et al. on injecting drug practices and HIV infection in Kigali identified bisexuality and initial needle sharing as significant risk factors associated with subsequent needle sharing and the transmission of blood-borne infections; therefore, the study advocated for increased implementation of harm-reduction practices [12]. However, to our knowledge, research on determinants associated with PWID in Rwanda remains limited. This gap may be partly related to the stigma and taboo surrounding injecting drug use, which is often perceived as illegal or socially unacceptable. Such perceptions can marginalize PWID and create barriers to their social inclusion and coexistence with the broader population [2]. This study aimed to explore the relationships among sociodemographic characteristics, substance and alcohol dependence, and depressive symptoms among PWID and to examine factors associated with PWID status within a sample at high risk for HIV/AIDS in Kigali, Rwanda. By addressing these relationships, this research seeks to fill critical gaps in understanding the complexities surrounding drug-use behaviors and their psychosocial implications within this context.

## Materials and methods

### Design and settings

This cross-sectional study examined multiple interrelated factors, including sociodemographic characteristics, substance and alcohol dependence, and depressive symptoms among key marginalised populations engaged in substance use and high-risk behaviours associated with HIV/AIDS in Kigali, Rwanda. Data were collected at two sites of the Rwandan Health Development Initiative (HDI): the HDI headquarters and Nyakabanda Health Clinic, both located in Kigali. A total of 480 surveys were administered to individuals classified as hard-to-reach populations and substance users within Kigali City. Of these participants, 151 were classified as people who inject drugs (PWID), while 329 did not meet this classification.

### Participants and sampling

Participants were eligible if they were aged 18 years or older, resided in Kigali, self-identified as MSM, FSW, PWID, or other substance users, and had no prior formal clinical diagnosis of depression (e.g., by a treating clinician). This exclusion criterion did not exclude participants who subsequently endorsed depressive symptoms on the Beck Depression Inventory-II (BDI-II) administered as part of the study. Because of the criminalisation and cultural stigmatisation of these behaviours within Rwandan communities, these groups were considered hard-to-reach populations. Consequently, a non-probability sampling approach using snowball, chain-referral, or referral sampling was employed. Participants were recruited through networks of trusted outreach workers who facilitated access to potential respondents within their social networks. For this study, PWID were operationally defined as individuals who self-reported injecting any illicit drug within the previous 12 months. “Other substance users” referred to participants who reported non-injection use of alcohol, marijuana, cocaine, diazepam, or cigarettes without a history of injection drug use.

### Data collection procedures and measures

Research assistants and nurses from the Rwandan Health Development Initiative (HDI) at two sites, including the HDI headquarters and the Nyakabanda Health Clinic, both in Kigali, were trained to administer a structured questionnaire in Kinyarwanda to study participants. Each participant was assigned a unique identification number to protect confidentiality. Participants received US $5 as compensation for their time and travel expenses. The questionnaire took approximately 15 minutes–30 min to complete.

#### Self-demographic questionnaire (SDQ)

The demographic variables examined in this study included age, marital status, gender, sexual orientation and behavior, relationship status, history of sex work, education, and employment status. Other variables included the type of injecting drug used, frequency of drug injection, HIV/AIDS status, and use of other substances. In total, 25 variables were identified.

#### Beck depression inventory, second edition (BDI-II)

The BDI-II is a psychometric instrument used to assess the severity of depressive symptoms and has previously demonstrated strong psychometric properties in both general and clinical populations in Rwanda. The BDI-II contains 21 items rated on a Likert scale from 0 to 3, with a maximum possible score of 63. A score below 14 indicates minimal depressive symptoms, a score from 14 to 19 indicates mild depressive symptoms, a score from 20 to 28 indicates moderate depressive symptoms, and a score ≥29 indicates severe depressive symptoms [26]. Cronbach’s alpha (α) in this study was 0.898.

#### The mini-international neuropsychiatric interview (MINI)

The MINI is a short structured diagnostic interview developed jointly by psychiatrists and other US and European clinicians for DSM-IV and ICD-10 psychiatric disorders. With an administration time of approximately 15 min, it was designed to meet the need for a short but accurate structured psychiatric interview for multicenter clinical trials and epidemiology studies and be used as the first step in outcome tracking in nonresearch clinical settings [13]. The MINI is divided into eight modules identified by letters, each corresponding to a diagnostic category. For this study, we used two such categories: alcohol dependence or abuse, with 21 items, and substance dependence or abuse, with 12 items. Each question asked about activities performed over 12 months, with response options being true or false, with true items indicating that a participant has symptoms of either alcohol or substance dependence and abuse or both. The Cronbach’s alpha (α) values for the alcohol and substance dependence scales were 0.915 and 0.793, respectively.

#### HIV testing

During the informed consent process, participants were informed that the study would include a rapid HIV test. The study participants underwent pretest and posttest counseling by research assistants trained in HIV counselling, testing, and referrals. Participants who received an initial positive result on the rapid HIV test were confirmed with a second rapid test. Two positive results were required for subjects to be classified as HIV positive. The participants who had discordant results between the two tests were classified as HIV negative. Those who had a previous HIV/AIDS diagnosis and were receiving treatment were only tested for HIV once.

### Data analyses

All participant data were collected and initially entered into Microsoft Excel for preliminary organization. Following data-cleaning procedures to ensure accuracy and consistency, the dataset was imported into IBM SPSS Statistics (version 28, 2019) for further statistical analysis. First, descriptive statistics were used to assess the sociodemographic characteristics of the sample, with results presented as frequencies and percentages. To examine potential associations, Pearson’s chi-square test (χ^2^) was used to determine statistically significant relationships between PWID status and other categorical variables, such as depressive symptoms, alcohol and substance use, and sociodemographic characteristics.

Multicollinearity among the independent variables was assessed using variance inflation factor (VIF) and tolerance values. The data did not violate these assumptions; therefore, all selected variables were retained for the final model. Subsequently, all variables that showed significant associations with PWID status in the bivariate analyses were included in a multivariate logistic regression model. This approach allowed calculation of adjusted odds ratios (aORs) to identify factors independently associated with PWID status while controlling for potential confounders. A 95% confidence interval (CI) and a 5% significance level (p < 0.05) were applied throughout the analyses.

## Results

### Descriptive analysis by sociodemographic variables

Our results were from the data from 480 participants, of whom 151 (31.5%) were identified as PWID. A segment of participants (12%) reported engagement in sex work as FSWs, whereas 57.9% were classified as MSM. Notably, 89.4% of PWID were single, compared with 72.3% of non-PWID participants. Among PWID, a majority (52.3%) were between 18 years and 25 years of age, and 93.4% were male. Bivariate analyses revealed statistically significant associations between PWID status and several demographic factors, including age (χ^2^ = 7.70, p = 0.050), sex (χ^2^ = 16.35, p < 0.001), MSM status (χ^2^ = 24.2, p < 0.001), FSW status (χ^2^ = 6.56, p = 0.006), marital status (χ^2^ = 19.38, p < 0.001), and education (χ^2^ = 8.20, p < 0.01). However, employment status (χ^2^ = 3.51, p = 0.30) was not significantly associated with injecting drug use (Table 1).

**TABLE 1:** Description of the sociodemographic characteristics o**f** PWID and bivariate associations of variables of interest with PWID (n = 480).

​	Descriptive analysis		Bivariate analysis	
Variables	PWID		Chi-square	p-value
​	Yes (151)	No (329)	​	​
Age (in years)	​	​	7.70	0.050*
18–25 years	79 (52.3)	140 (42.6)	​	​
26–35 years	59 (39.1)	132 (40.1)	​	​
36–45 years	11 (7.3)	45 (13.7)	​	​
45+ years	2 (1.3)	12 (3.6)	​	​
Gender	​	​	16.35	<0.001***
Male	141 (93.4)	280 (85.1)	​	​
Female	10 (6.6)	49 (14.9)	​	​
Relationship status	​	​	19.38	<0.001***
Single	135 (89.4)	238 (72.3)	​	​
Married	9 (6.0)	41 (12.4)	​	​
Divorced/Widower	7 (4.6)	50 (15.2)	​	​
FSW	​	​	6.56	0.006**
Yes	10 (6.6)	49 (14.4)	​	​
No	141 (93.4)	280 (85.6)	​	​
MSM	​	​	24.2	<0.010**
Yes	110 (72.8)	160 (47.1)	​	​
No	41 (27.2)	169 (52.9)	​	​
Education	​	​	8.20	<0.010**
None	4 (2.6)	12 (3.6)	​	​
Primary	28 (18.5)	100 (30.4)	​	​
Secondary	119 (78.8)	217 (66.0)	​	​
Employment status	​	​	3.51	0.30
Unemployed	54 (35.8)	93 (28.3)	​	​
Student	24 (15.9)	48 (14.6)	​	​
Employed	15 (9.9)	37 (11.2)	​	​
Other	58 (38.4)	151 (45.9)	​	​

*statistical significance at p < 0.05; ** statistical significance at p < 0.01; *** statistical significance at p < 0.001, Yes: Number of injecting drug users; No: Number of non -injecting drug users.

### Description of substance and alcohol use and their associations with PWID

Table 2 shows that, among PWID, 73.5% reported alcohol use and 61.6% reported marijuana use. Notably, 96.0% of PWID exhibited symptoms of drug dependence, whereas 44.5% met the criteria for alcohol dependence or abuse. Overall, depressive symptoms were highly prevalent in the sample. HIV prevalence was 2.6% among PWID and 5.7% among non-PWID participants. Regarding prior substance-use treatment, 29.1% of PWID, compared with 14.3% of non-PWID participants, reported having sought treatment. Depressive symptoms were reported by 94.0% of PWID. Bivariate analyses revealed statistically significant associations between injection drug use and alcohol consumption (wine, hard alcohol, and liquor) (χ^2^ = 25.07, p < 0.001), cigarette smoking (χ^2^ = 5.80, p < 0.01), marijuana use (χ^2^ = 3.77, p = 0.050), and diazepam use (χ^2^ = 11.90, p < 0.001). Moreover, individuals who injected drugs were more likely to have sought treatment for substance use (χ^2^ = 14.86, p < 0.001) and to exhibit depressive symptoms (χ^2^ = 17.76, p < 0.001). Although 4.7% of the sample tested positive for HIV, there was no statistically significant association between HIV status and PWID status (χ^2^ = 2.21, p = 0.136). Similarly, family history of substance abuse was not significantly associated with PWID status in the sample (χ^2^ = 0.98, p = 0.320) (Table 2).

**TABLE 2:** Description of the substance use variables comparin**g** PWID and other high-risk groups for HIV/AIDS and bivariate associations of drug use variables with PWID.

​	Descriptive analysis		Bivariate association analysis	
Variables	PWID		Chi-square	p value
​	Yes, N (%)	No, N (%)	​	​
Used alcohol	​	​	25.07	<0.001***
Ever	111 (73.5)	299 (90.9)	​	​
Never	40 (26.5)	30 (9.1)	​	​
Alcohol dependence	​	​	24.06	<0.001***
Yes	65 (44.5)	234 (71.1)	​	​
No	81 (45.5)	95 (28.9)	​	​
Drug dependence	​	​	13.00	<0.001***
Yes	145 (96.0)	262 (84.5)	​	​
No	6 (4.0)	48 (15.5)	​	​
Used cigarettes	​	​	5.80	<0.001**
Ever	125 (82.8)	239 (72.6)	​	​
Never	26 (17.2)	90 (27.4)	​	​
Marijuana	​	​	3.77	0.050
Ever	93 (61.6)	232 (70.5)	​	​
Never	58 (38.4)	97 (29.5)	​	​
Diazepam	​	​	11.90	<0.001***
Ever	28 (22.2)	31 (9.8)	​	​
Never	98 (77.8)	284 (90.2)	​	​
Cocaine	​	​	2.97	0.080
Ever	7 (6)	8 (2.6)	​	​
Never	110 (94)	305 (97.4)	​	​
Sought treatment	​	​	14.86	<0.001***
Yes	44 (29.1)	47 (14.3)	​	​
No	107 (70.9)	282 (85.7)	​	​
Family history	​	​	0.98	0.320
Yes	94 (62.3)	191 (58.1)	​	​
No	57 (37.7)	138 (41.9)	​	​
Depression status	​	​	17.76	<0.001**
Yes	142 (94.0)	271 (82.3)	​	​
No	9 (6.0)	57 (11.7)	​	​
H.I.V. status	​	​	2.21	0.136
Yes	4 (2.6)	19 (5.7)	​	​
No	147 (97.4)	310 (94.2)	​	​

*Statistical significance at p < 0.05; ** statistical significance at p < 0.01; *** statistical significance at p < 0.001, Yes: Number of injecting drug users; No: Number of non -injecting drug users.

### Multivariate logistic regression model analyses for the associated factors for PWID

The multivariate logistic regression analysis indicated that participants with substance dependence disorders were almost four times more likely to be PWID (aOR = 3.547; 95% CI [1.159–10.857], p = 0.027) than their counterparts. Conversely, participants with alcohol dependence had lower odds of being PWID (aOR = 0.511; 95% CI [0.285–0.918], p = 0.028). MSM status was significantly associated with increased odds of being PWID (aOR = 3.20; 95% CI [1.816–5.640], p < 0.001). Female participants had lower odds of being PWID than male participants (aOR = 0.698; 95% CI [0.248–0.967], p = 0.031). Marijuana users were more likely to be PWID than nonusers (aOR = 3.261; 95% CI [1.380–7.708], p = 0.007), whereas cocaine users were less likely to be PWID than their counterparts (aOR = 0.404; 95% CI [0.184–0.887], p = 0.024). Participants with depressive symptoms were more likely to be PWID than those without depressive symptoms (aOR = 4.50; 95% CI [2.55–7.95], p = 0.018). Variables such as age, cigarette use, education level, family history of substance use, and marital status were not associated with the likelihood of being PWID. These findings provide insight into the interplay of mental health, substance dependence, and demographic factors associated with PWID status within this specific context (Table 3).

**TABLE 3:** Multivariate logistic regression model analyses for the risk factors for PWID.

​	Adjusted odds ratio	95% confidence intervals		​
Variables		Lower	Upper	P values
Age				
18–25 years	1	​	​	​
26–35 years	0.635	0.282	1.431	0.273
6–45 years	0.996	0.416	2.384	0.994
45+ years	0.766	0.281	2.09	0.603
Education				
None	1	​	​	​
Primary	1.327	0.236	7.47	0.748
Secondary and above	0.961	0.172	5.356	0.964
Gender				
Male	1	​	​	​
Female	0.698	0.248	0.967	0.031*
Use of alcohol				
No	1	​	​	​
Yes	0.487	0.275	0.862	0.014*
M.S.M.				
No	1	​	​	​
Yes	3.2	1.816	5.64	<0.001**
Heroin users				
No	1	​	​	​
Yes	0.697	0.377	1.291	0.479
Cigarette				
No	1	​	​	​
Yes	0.892	0.406	1.963	0.777
Marijuana				
No	1	​	​	​
Yes	3.261	1.38	7.708	0.007*
Diazepam				
No	1	​	​	​
Yes	1.277	0.649	2.515	0.777
Cocaine				
No	1	​	​	​
Yes	0.404	0.184	0.887	0.024*
Alcohol dependence				
No	1	​	​	​
Yes	0.511	0.285	0.918	0.028*
Substance dependence				
No	1	​	​	​
Yes	3.547	1.159	10.857	0.027*
Sought treatment				
No	1	​	​	​
Yes	1.798	0.916	3.529	0.088
Family history				
No	1	​	​	​
Yes	0.728	0.41	1.292	0.278
Depression				
No	1	​	​	​
Yes	4.50	2.55	07.95	0.018*

*Statistical significance at p < 0.05; ** statistical significance at p < 0.01; *** statistical significance at p < 0.001, Yes: Number of injecting drug users; No: Number of non -injecting drug users.

## Discussion

The findings of this study revealed a high prevalence of depressive symptoms among participants at high risk for HIV/AIDS in Kigali, Rwanda. Overall, 86.25% of participants reported depressive symptoms, and most PWID had moderate to severe symptoms. A 2015 study by Li et al. in China supports our findings, showing that injecting drug use is closely associated with depression and other mental health problems [27]. Depressive disorders are commonly associated with addiction. Some people may inject drugs while experiencing depression, whereas others report that injecting drug use contributes to depressive symptoms [21, 27, 28]. The high prevalence of depressive symptoms in Rwanda is not a new phenomenon. The post-genocide period in Rwanda has been characterized by a high prevalence of depression within the community. Recent studies have shown that poor mental health is common across demographic groups in Rwanda [23, 25]. Nevertheless, the prevalence of depressive symptoms among PWID (94%) and among participants in other groups at risk for HIV/AIDS (82.3%) far exceeds previously reported rates of 35% among genocide survivors and 20% in the general population [23].

Our study revealed that participants’ age and sexual orientation were associated with PWID status in the bivariate analyses. For example, younger participants and sexual minorities, particularly MSM and FSW, were more frequently represented among PWID. Our findings align with previous research conducted in Africa, including a study in Kenya that documented heroin injection among young women engaged in sex work and men from sexual minority groups [28].

Regarding mental health, our results showed that participants with depressive symptoms were more likely to be PWID than those without depressive symptoms. This finding is consistent with the self-medication hypothesis, whereby the co-occurrence of depressive symptoms and limited access to mental health resources in low-income settings may be associated with substance use, including injection drug use, as a coping strategy [9]. However, given the cross-sectional design, the reverse pathway is equally plausible: substance-induced depression, such as during withdrawal from opioids or stimulants or as a neuroadaptive consequence of chronic use, is well documented in the neuropsychopharmacology literature, and injecting drug use may itself contribute to depressive symptoms rather than result from them. Moreover, the illegality and stigma surrounding injecting drug use may discourage individuals with co-occurring depressive symptoms from seeking mental health assistance. This interplay between depression and limited mental health support in Rwanda may heighten vulnerability to injecting drug use as a means of coping. These findings are consistent with previous studies [29].

As this was a cross-sectional study, we cannot determine which behavior occurred first. It is often assumed that individuals using other drugs may progress to injection drug use over time. Thus, it is not surprising that substance-use behaviors, particularly marijuana use, were associated with PWID status. These findings align with an earlier study conducted in China among women who injected drugs and also engaged in sex work. Those studies highlighted that these women had a greater likelihood of experiencing suicidal ideation, engaging in risky behaviors, and using alcohol and other substances [21, 30]. Clients of sex workers provided them with drugs and frequently shared injecting equipment. Corroborating these findings, a survey conducted in the Saskatoon Health Region in Canada revealed that initiation of substance use often begins in childhood and is influenced by networks of PWID, including friends and schoolmates [31]. Notably, Rwanda has a predominantly young population, and many participants in our study were younger than 25 years. These results indicate substantial substance use among younger participants, although age at initiation was not explicitly explored in our research.

A recent study of injecting drug practices and HIV/AIDS among PWID revealed that 99% of PWID primarily used heroin, 91% had shared needles in their lifetime, and 31% had shared needles in the previous 6 months. HIV prevalence was 9.5%, and needle sharing and other risky behaviors, such as reusing needles and sharing drug paraphernalia, significantly increased risk, particularly among women in Rwanda [12, 25]. We also found that depressive symptoms, alcohol and substance dependence disorders, and a history of seeking substance-use treatment were significantly associated with injection drug use. These findings are consistent with previous studies conducted among fisherfolk in China and other groups in Vietnam, Tanzania, and Kenya, which showed that adolescents of both sexes and sexual minorities, including FSW and MSM, are more likely to engage in injection drug use [21, 27, 30]. A recent study of injecting drug use in Rwanda by Rwema et al. (2022) established an association between injecting drug use and HIV/AIDS. However, that study did not examine how depressive symptoms were associated with injection drug use. Our findings indicate that a considerable proportion of Rwandan youth who inject drugs report depressive symptoms. Such behavior may serve as a coping mechanism to escape or alleviate depressive mood.

Although substance use was high overall, our results indicated that PWID in our sample were more likely to meet criteria for drug dependence than for alcohol dependence. Nevertheless, both drug and alcohol dependence were associated with injecting drug use. Drug use may progress from alcohol or non-injection drug use to injection drug use over time. PWID may use other types of drugs through different routes, including sniffing cocaine and using marijuana, in addition to alcohol use [9, 15].

Finally, 4.7% of participants in our study were HIV positive. This finding indicates that our sample, which was recruited because it included individuals at greater risk for HIV/AIDS, had a higher HIV prevalence than the approximately 3% reported among Rwandan adults aged 15 years –49 years [32]. However, the somewhat unexpected finding that HIV prevalence among PWID was lower (2.6%) than among the other risk groups in the study (5.7%) warrants cautious interpretation rather than reassurance. Because PWID in our sample were significantly younger than non-PWID participants (52.3% vs. 42.6% aged 18–25 years), the lower observed HIV prevalence among PWID may reflect shorter cumulative exposure time rather than a true reduction in risk. Age-stratified or age-adjusted comparisons are needed to disentangle these effects before concluding that intervention has averted HIV acquisition in this group; we identify this as a priority for further analysis (see Limitations).

Two previous studies of HIV-positive PWID reported a high prevalence of depressive symptoms in this group, ranging from 42% to 44% [33, 34], which was lower than the prevalence observed in our study. Both studies identified depression as a factor associated with PWID among HIV-positive individuals. Depressive symptoms and mental health disorders are not new among people who use illicit drugs; however, the types of substances used can affect symptom severity. For example, research conducted in Mwanza, Tanzania, revealed that heroin users were more likely to engage in unprotected sex, thereby increasing exposure to HIV/AIDS and other blood-borne diseases, which can contribute to depression [35].

Our findings differ from the prevailing literature, as family background was not statistically associated with injecting drug use. This contrasts with European research findings in which more than half of participants reporting frequent domestic conflicts had a family history of substance use, and three-fourths had peers engaged in injecting drug use [36]. The absence of such associations in our results may be attributable to the relatively underexplored nature of injecting drug use, which represents a rapidly evolving trend in African countries, including Rwanda. Compared with other forms of substance use, injecting drug use is a relatively new phenomenon in this context, and familial influences on this specific route of drug administration are not yet well understood.

In contrast to the patterns observed for injecting drug use in this study, family members in Rwanda may influence their offspring in matters related to substance use [37]. Substance use by a family member may affect a child through mechanisms such as increased genetic predisposition or negative role modelling, thereby heightening the child’s susceptibility to substance use [38]. In Rwandan culture, as in many other societies, children may emulate behaviors demonstrated by their parents, and substance use, including drinking and smoking, can reflect a familial culture of use.

### Study strengths and limitations

This study has several important strengths. To our knowledge, it is the first investigation of the relationship between mental health and PWID status in Kigali, Rwanda. Notably, the study revealed a high prevalence of depressive symptoms among people at risk for HIV/AIDS and identified a significant association between depressive symptoms and PWID status. Additionally, it identified vulnerable populations, including younger individuals and sexual minorities, and highlighted their greater representation among PWID. The study’s focus on behavioral influences, particularly alcohol and substance use, extends our understanding of these factors in relation to PWID patterns. Furthermore, these findings have important implications for tailored interventions and public health strategies, emphasizing the need to expand mental health services, particularly among vulnerable groups. These insights may inform targeted interventions to mitigate risks associated with injecting drug use and improve mental health outcomes in Rwanda and similar contexts in sub-Saharan Africa.

Despite the strengths of this study, we acknowledge several limitations. First, owing to the cross-sectional study design, we were unable to determine a causal relationship between various types of drugs and alcohol exposure and PWID. Clearly, further studies using a longitudinal study design are essential for allowing us to assess the progression of risk factors, including depression, among PWID. Second, the sample size was somewhat limited, as it was derived through nonrandom, snowball sampling because the study population is hard to reach because of the hidden and illegal nature of the behavior. This could result in an overestimation or underestimation of our results, which prevents us from generalizing. Future studies should employ larger sample sizes and some type of more systematic recruitment, as randomization is clearly never possible.

The study findings highlight the high prevalence of depressive symptoms in our sample of individuals at high risk for HIV/AIDS, with more than 86.25% of participants exhibiting depressive symptoms. Depression was even more common among PWID (94%). The observed relationship between PWID status and depressive symptoms aligns with previous global research linking depressive disorders with alcohol and substance use disorders. These findings emphasize the need for tailored interventions addressing depressive symptoms, especially among vulnerable populations such as sexual minorities (MSM and FSW) and individuals engaging in substance use, particularly hard alcohol and marijuana use. The finding that family background was not statistically associated with PWID status contrasts with established patterns in other forms of substance use and may reflect the evolving nature of injecting drug use in Rwanda and the limited exploration of familial influences on this specific route of drug administration.

Moreover, because we limited the line of questioning to keep the survey brief and optimise response rates, we were unable to explore potentially important sociocultural, structural, and belief-related factors. Furthermore, the use of self-reported measures may have introduced recall bias, which could affect the reliability of the data. Therefore, at the very least, mixed methods could be important for exploring all possible factors of PWID. This could then be followed by a longer survey aligned with the contextual findings. In addition, in the process of gathering data on alcohol and substance use, we utilized the MINI, which employs DSM-IV criteria. However, certain criteria were modified and consolidated in the updated DSM-V version, potentially leading to misunderstandings in data concerning alcohol and substance dependence. Additionally, recruitment occurred through outreach linked to a single NGO service provider (Health Development Initiatives) at two sites, which may over-represent individuals already connected to services and underrepresent hidden populations of PWID who avoid such services, a further source of selection bias beyond the snowball sampling method itself. Both PWID status and depressive symptoms were determined solely by self-report, without objective verification (e.g., urine toxicology or collateral informant report); illegal and stigmatized behaviors such as injection drug use are particularly susceptible to social desirability bias, which may have led to under- or over-reporting in either direction. We also did not report the response or refusal rate among individuals approached for participation, which limited our ability to assess the potential for non-response bias. Finally, the BDI-II is a validated screening measure of depressive symptom severity and validated in Rwanda [39] and does not constitute a clinical diagnosis of major depressive disorder; our use of the term “depression” throughout refers to symptom-level screening results rather than a formal diagnosis.

### Implications for practice

Moving forward, addressing mental health challenges related to depressive symptoms and substance dependence among youth who inject drugs is increasingly important. Mental health services should prioritize the co-occurring needs related to depressive symptoms, alcohol addiction, and substance use, particularly given that only a small proportion of Rwandans seek help for mental health concerns. Given the above-mentioned limitations, future research should include mixed-methods studies to further explore the context and progression of injecting drug use, followed by larger, adequately powered longitudinal studies that can help clarify causal relationships. Moreover, as this research focused on urban areas in Kigali city, future research should explore other geographic areas beyond Kigali to better understand the distribution and contextual factors associated with injecting drug use across Rwanda. Larger-scale studies involving varied populations would also enhance understanding and generalizability. Representative and repeated assessments of substance use, mental health, and health-risk behaviors across Rwanda could further elucidate the complex interplay among depressive symptoms, substance use, and health-risk behaviors among PWID in Rwanda and neighbouring sub-Saharan African countries.

## Conclusion

This study provides important evidence on the intersection of depressive symptoms, substance use, and injecting drug use among individuals at high risk for HIV/AIDS in Kigali, Rwanda. A high prevalence of depressive symptoms was observed, particularly among PWID, and depressive symptoms, alcohol and substance dependence, and previous treatment for substance use were significantly associated with PWID status. Younger participants and sexual minority groups, including MSM and FSW, were also more frequently represented among PWID. These findings highlight the overlapping mental health, substance-use, and social vulnerabilities associated with injecting drug use in this setting.

Given the cross-sectional design, these associations should not be interpreted as causal relationships. The results from this work nevertheless underscore the importance of integrated health services that address mental health, substance use, and HIV-related vulnerabilities, particularly among younger and socially vulnerable populations. Future longitudinal and mixed-methods research involving larger and more diverse populations, including settings beyond Kigali, is needed to clarify the temporal relationships among depressive symptoms, substance use, injecting drug use, and HIV-related risks. Such evidence can support the development of contextually responsive harm-reduction and mental health strategies for PWID in Rwanda.

## Data Availability

The raw data supporting the conclusions of this article will be made available by the authors, without undue reservation.
